# Optimization of 3D Printing Nozzle Parameters and the Optimal Combination of 3D Printer Process Parameters for Engineering Plastics with High Melting Points and Large Thermal Expansion Coefficients

**DOI:** 10.3390/ma18030500

**Published:** 2025-01-22

**Authors:** Jun Wang, Hang Hu, Ziyi Liu, Yuanyuan Shi, Yizhe Huang

**Affiliations:** 1 Hubei Key Laboratory of Modern Manufacturing Quality Engineering, School of Mechanical Engineering, Hubei University of Technology, Wuhan 430068, China; junwang@hbut.edu.cn (J.W.); 102200018@hbut.edu.cn (H.H.); 2011311217@hbut.edu.cn (Z.L.); 2 State Key Laboratory of Digital Manufacturing Equipment and Technology, Huazhong University of Science and Technology, Wuhan 430074, China; d201980200@hust.edu.cn

**Keywords:** 3D printing, nozzle structure, fluid simulation, process parameters, orthogonal experiments, molding accuracy

## Abstract

Three-dimensional printing is a transformative technology in the manufacturing industry which provides customization and cost-effectiveness for all walks of life due to its fast molding speed, high material utilization, and direct molding of arbitrary complex structural parts. This study aims to improve the molding accuracy of 3D printed polyether ether ketone (PEEK) samples by systematically studying key process parameters, including printing speed, layer thickness, nozzle temperature, and filling rate. The 3D printing nozzle has an important impact on the extrusion rate of the melt, and the fluid simulation of the nozzle was carried out to explore the variation characteristics of the melt flow rate in the nozzle and optimize the nozzle structure parameters. In order to effectively optimize the process, considering its inherent efficiency, robustness, and cost-effectiveness, the L9 orthogonal array experimental design scheme was used to analyze the effects of printing speed, layer thickness, nozzle temperature, and filling rate on the molding accuracy of the test sample, and the optimal combination of process parameters was optimized through the comprehensive weighted scoring method so as to improve the molding accuracy of the 3D printed PEEK sample; finally, the molding accuracy of the components printed using the Sermoon-M1 3D printer with the optimized nozzle structure was printed. The results show that the nozzle structure is optimal when the convergence angle is 120° and the aspect ratio is 2, and the outlet cross-section velocity is increased by 2.5% and 2.7%, respectively. The order of influence strength on the dimensional accuracy of the test sample is layer thickness > filling rate > nozzle temperature > printing speed. The optimal combination of parameters is: a printing speed of 15 mm/s, a layer thickness of 0.1 mm, a nozzle temperature of 420 °C, and a filling rate of 50%. The insights derived from this study pave the way for predicting and implementing the selection of optimal process parameters in the production of 3D printed products, with important implications for the optimal molding accuracy of printed components.

## 1. Introduction

Among the many 3D printing technologies, fused deposition modeling (FDM) is one of the most rapidly developed and widely used technologies which not only has the general advantages of 3D printing technologies, but also has its own advantages such as low equipment manufacturing cost, simple technical principles, and pollution-free green manufacturing [1,2]. At present, the materials widely used in the FDM process are basically low-melting polymers, such as polylactic acid (PLA), acrylonitrile–butadiene–styrene (ABS), polycarbonate (PC), paraffin, etc. [3,4,5]. In recent years, special engineering plastics have been favored in the biomedical, aerospace, and defense industries and in automobile manufacturing due to their unique comprehensive properties, such as polyether ether ketone (PEEK), polyetherimide (PEI), and polyphenylene sulfide (PPS), which can work for a long time in harsh environments [6,7,8,9]. With the rapid development of science and technology, people have more complex, lightweight, and integrated requirements for the workpiece structure made of high-performance materials, and the traditional polymer processing technology cannot meet the current production requirements. FDM 3D printing technology provides a solution to this by virtue of its own characteristics, but the FDM 3D printing machines on the market are mainly suitable for the traditional low-melting-point polymer materials, and the research on the molding equipment for special engineering plastics with a high melting point and a large expansion coefficient is in the development stage. The study of the performance of the equipment and the molding process still needs to be deepened. Therefore, based on the existing FDM technology, this paper develops a high-temperature FDM 3D printing device with independent intellectual property rights, a high cost–performance ratio, and high reliability. This device can not only print high-performance special engineering plastics (such as PEEK, PEI, PPSU, etc.), but also takes into account the commonly used materials on the market (such as PLA, ABS, PC, etc.).

During the printing process, the printing components are easily affected by factors such as printing speed, nozzle, layer thickness, and printing temperature. The nozzle and layer thickness are the main factors influencing printing accuracy, and the stability of the nozzle outlet speed, viscosity, and outlet pressure are important indicators reflecting printing accuracy [10]. Therefore, it is of great importance to conduct research on the structure of nozzles. Joko [11] experimentally studied the influence of the nozzle aperture in 3D printing on the surface quality, accuracy, and strength of products. Polylactic acid (PLA) was selected as the manufacturing sample, ensuring that the ratio of the printing layer thickness to the nozzle aperture was 20%. Other factors such as printing temperature and printing speed were kept constant. The results showed that the larger the nozzle aperture, the higher the density and tensile strength of the products, but there was no linear correlation between them. While the quality and strength of the product are sufficiently high, it is also required that the precision and dimensions of the product be accurate enough to meet the engineering needs. Yu [12] utilized the COMSOL multiphysics fluid-solid coupling to conduct a simulation analysis on the structural characteristics of the printer nozzle. A combination of two-phase flow was adopted to simulate the morphological characteristics of the melt flowing out of the nozzle, and an orthogonal experiment was designed with the melt outlet speed, stability, viscosity, and outlet pressure as indicators. The results showed that, as the nozzle diameter decreased, the melt pressure decreased, the speed increased, and the viscosity decreased. The outlet diameter of the nozzle was inversely proportional to the printing accuracy. The research results provide a theoretical reference for the optimal selection of nozzle apertures. Han [13] extracted and simplified the flow channel model of the nozzle. CAD and finite element models were established through the UG and ICEM CFD software. The flow field was simulated by the Fluent software to obtain the suitable temperatures of the nozzle under different extrusion speeds. Finally, the nozzle was tested and verified. It could stably generate color-mixing artifacts, verifying the rationality of the structure of the nozzle of the color-mixing fused deposition modeling 3D printer. At present, there are relatively few optimization studies on the nozzle structure on the market. Most of them focus on the monitoring research of nozzle blockage and printing status, and the choice of nozzle structure mainly relies on experience, making the research on nozzle structure challenging [14,15]. Here, most of the scholars’ analyses focus on the single-factor variable of the nozzle aperture, while there is relatively little research on the quantitative relationship between the nozzle aperture and the length of the discharge section in terms of printing precision.

There are many types of printing test materials available on the market. Moreover, the structural characteristics of nozzles result in different dimensional precision and mechanical properties of the samples printed with different materials. The following scholars have carried out research on different materials. Wang [16] studied the influence of the changes in the width and layer height of the extruded material on the bending performance when the nozzle diameter of the high-extrusion-rate fused deposition 3D printer was increased from 0.4 mm to 1.0 mm. Short carbon-fiber-filled polyamide 12 (PA12-CF) was used as the test material. The results showed that, with the increase in layer height and extrusion width, the printed samples exhibited better mechanical properties, with the bending strength and stiffness increasing by 20% and 30%, respectively. Czyżewski [17] employed RepRap equipment and nozzles with diameters of 0.2 mm, 0.4 mm, 0.8 mm, and 1.2 mm. Under the condition that the layer height was 0.2 mm and the filling rate was 100%, comparisons were made with the inherent structural samples produced by injection molding technology to study the impact of the diameter of the extruder nozzle on the functional characteristics of 3D printed PLA products. The results indicated that favorable mechanical properties could be obtained by using a layer height lower than the standard and a nozzle diameter of 0.8 mm. However, the use of nozzles with larger diameters would result in the accumulation of excess materials due to poor distribution, which would then reduce the surface quality and stiffness. The shortening of the manufacturing time was accompanied by a decrease in printing accuracy and changes in the viscous behavior of the materials. Da Silva [18] carried out parameter calibration for the cube model printed with polylactic acid (PLA) using a nozzle with a diameter of 1 mm through three steps: identifying printing defects, studying system parameters, and optimizing parameters interactively. Six variables including layer thickness, extrusion temperature, extrusion speed, filling speed, perimeter speed, and the speed of the first layer were analyzed. Factors such as the aesthetic quality, dimensional and shape consistency, mass, and changes in compressive modulus of the parts were taken into consideration. The results showed that a nozzle with a larger diameter was not “plug-and-play”, and it needed to be determined according to the printability of the material and the correlation among the influencing variables involved in slicing. Extrusion speed and layer thickness were the main issues directly affecting printing with a 1 mm nozzle. Temperature is very important when manufacturing products in different scenarios. Thermal properties seriously affect the mechanical properties of products, and the temperature of the printing nozzle also significantly affects the mechanical properties of products. Ulkir [19] studied the influence of the change in the temperature of the printing nozzle on the size and mechanical properties of samples prepared from acrylonitrile butadiene styrene (ABS) material. The nozzle temperature was increased from 220 °C to 270 °C in increments of 10 °C. A total of 36 tensile test samples were manufactured by the fused filament fabrication method. Stress–strain values were measured through tensile testing, and an analysis of variance was used to examine the influence of the change in nozzle temperature on the experimental research results. The results showed that, at higher temperatures, both the mass and the density of the samples decreased with the increase in temperature, and the tensile strength decreased by 41.52%. Meanwhile, Grubbs [20] took PLA as a case study and established a multi-step process optimization method. The results showed that, under the optimal printing conditions, the component size and tensile properties would change according to the combination of nozzle temperature, forming platform conditions, filling settings, and annealing conditions. The research on printing test materials is mainly based on the commonly available materials on the market, while there is relatively little research on engineering plastics with high melting points and large thermal expansion coefficients.

The fused deposition modeling (FDM) process plays a decisive role in the mechanical properties, dimensional accuracy, and surface finish of the formed parts [21]. In view of the impacts of different process parameters and the variety of test materials, exploring the optimal combination of process parameters for different test materials is a crucial step that affects the forming accuracy and mechanical properties of samples made of different materials. For this reason, a large number of scholars have adjusted process parameters through different methods to improve the quality of the formed parts. Yang et al. [22] used the orthogonal experiment to analyze the influences of layer thickness, printing speed, nozzle temperature, and filling angle on the tensile strength and impact strength of the printed parts and obtained the optimal combination of process parameters through the comprehensive scoring method and the comprehensive balance method. Ding [23] optimized the FDM process parameters with ANSYS finite element analysis combined with actual experiments and measured that the mechanical properties of the printed honeycomb structural parts were more excellent. Dey et al. [24] adopted the particle swarm intelligence algorithm to conduct multi-objective optimization on layer thickness, printing direction, filling rate, and extrusion temperature, a process which improved the tensile strength of the formed parts and reduced the required printing time. Syrlybayev et al. [25] used the finite element analysis (FEA) model developed by them for simulation and combined it with experimental analyses. The results all showed that the warpage of the formed parts decreased with the increase in layer thickness. Bai et al. [26] established a multi-objective optimization model for the dimensional accuracy of parts by using the grey relational method on the basis of the results of the orthogonal experiment analysis and carried out experimental verification on the obtained optimal combination of process parameters. Li [27] and Wu et al. [28] both combined the genetic algorithm with the BP neural network and, respectively, established the prediction models for the dimensional accuracy and warpage deformation of FDM 3D printed parts. The accuracy of the GA-BP neural network model was proved through comparative experiments. Dong et al. [29] used the adaptive cuckoo search algorithm and the deep belief network to construct a prediction model for the complex nonlinear relationship between the quality characteristics of the formed parts and the process parameters and verified that the prediction accuracy rate of this model could reach above 96.67%. Yang [30] adopted the method combining single-factor and orthogonal experiments to study the influences of filling angle, printing speed, printing temperature, filling density, and layer thickness on the tensile strength of polylactic acid.

This paper aims to systematically study the nozzle structure and conduct research on the optimal combination of process parameters for polyether ether ketone (PEEK), an engineering plastic with a high melting point and a large thermal expansion coefficient, based on the Sermoon-M1 3D printer in a laboratory. Through finite element thermal fluid simulations, the influence of the nozzle’s length-to-diameter ratio and convergence angle on the internal melt extrusion speed is analyzed. The nozzle structure is optimized for PEEK, a special engineering plastic with a high melting point and a large expansion coefficient, and the optimized nozzle structure is applied to the Sermoon-M1 3D printer in a laboratory to analyze the optimal combination of process parameters. The orthogonal test is used to analyze the influence of printing speed, layer thickness, nozzle temperature, and filling rate on the forming accuracy. The comprehensive weighted scoring method is employed to obtain the optimal combination of process parameters and to test and verify the effectiveness of the optimization of the optimal process parameters. The author hopes that, in the production of 3D printed products using engineering plastics with high melting points and large thermal expansion coefficients, this research can provide guidelines for nozzle structure and pave the way for predicting and implementing the selection of optimal process parameters. It is of great significance for achieving the optimal forming accuracy of printing components.

## 2. Nozzle Flow Field Velocity Simulation and Structural Parameter Optimization

The 3D printing nozzle is a core component of the FDM 3D printing equipment, and its structure has a significant impact on the melt extrusion speed. In this section, based on the ANSYS Fluent finite element thermal fluid analysis, the velocity field of the inner flow channel of the nozzle is analyzed and, through single-factor experiments, the fluid velocity distribution under different convergence angles and length-to-diameter ratios is explored. Finally, a comparative analysis of the melt extrusion speed before and after the nozzle optimization is carried out.

### 2.1. Finite Element Theory of Thermal Fluid Simulation

According to the principle of fused deposition modeling, the printing consumables are heated to a molten state inside the nozzle and then extruded. This process involves both heat transfer and the three fundamental control theories of fluid mechanics.

#### 2.1.1. Basic Modes of Heat Transfer

The transfer of heat is caused by the temperature difference within an object or between objects. According to the second law of thermodynamics, heat always spontaneously transfers from a place with a higher temperature to a place with a lower temperature. Based on different heat transfer mechanisms, there are three basic ways of heat transfer: heat conduction, heat convection, and thermal radiation.

1.  Heat Conduction

Heat conduction refers to the heat transfer that occurs due to the thermal motion of microscopic particles such as molecules, atoms, and free electrons when there is no relative displacement among different parts of an object. The calculation formula for heat conduction can be described by Fourier’s law, and its formula is:

(1) $$\left\{\begin{array}{c} q=\frac{\Phi }{A}=-k\frac{dT}{dx}\\ \Phi =-kA\frac{dT}{dx} \end{array}\right.$$

In the formula, Φ represents the amount of heat transferred per unit time; q represents the amount of heat transferred through a unit area per unit time; A represents the cross-sectional area perpendicular to the direction of heat conduction; k represents the thermal conductivity coefficient of the material; and the negative sign indicates that the direction of heat conduction is opposite to the direction of the temperature gradient.

2.  Heat Convection

Heat convection refers to the heat transfer between a medium (liquid or gas) and the solid surface when the medium flows over the solid. The calculation formula for heat convection can be expressed by Newton’s law of cooling, and its formula is:

(2) $$\left\{\begin{array}{c} q=\frac{\Phi }{A}=h\left(T_{w}-T_{f}\right)\\ \Phi =hA\left(T_{w}-T_{f}\right) \end{array}\right.$$

In the formula, h represents the convective heat transfer coefficient; $T_{w}$ and $T_{f}$ represent the temperature of the solid surface and the temperature of the medium, respectively.

3.  Thermal Radiation

Thermal radiation refers to the process in which electromagnetic waves are generated by a substance due to its own temperature and then absorbed by another low-temperature object, and then are completely or partially converted back into thermal energy again. The difference between thermal radiation and the previous two heat transfer methods is that the first two require the existence of substances, while thermal radiation can transfer energy in a vacuum and has the highest transfer efficiency in a vacuum. The radiative heat flux of an object can be calculated according to the empirical formula of the Stefan–Boltzmann law:

(3) $$q=\varepsilon A\sigma T^{4}$$

In the formula, ε represents the emissivity coefficient of the object; A  represents the surface area subjected to thermal radiation; σ represents the Stefan–Boltzmann constant, which is approximately $5.67032\times 10^{-8}\;W/\left(m^{2}K^{4}\right)$; and T represents the absolute temperature.

#### 2.1.2. The Governing Equations of Fluid Flow

1. The Mass Conservation Equation

The mass conservation equation is also called the continuity equation. Its physical meaning is that the increase in the mass of the fluid within a differential element per unit time is equal to the net mass flowing into this differential element in the same time interval. Its governing equation is:

(4) $$\frac{𝜕\rho }{𝜕t}+\frac{𝜕\left(\rho u_{x}\right)}{𝜕x}+\frac{𝜕\left(\rho u_{y}\right)}{𝜕y}+\frac{𝜕\left(\rho u_{z}\right)}{𝜕z}=0$$

In the formula, ρ represents the fluid density; t represents time; $u_{x}$,$u_{y}$, and $u_{z}$, respectively, represent the velocity components of the fluid in the x, y, and z directions. In this paper, the fluid simulation object is polyether ether ketone (PEEK) material, which is an incompressible fluid in the molten state, so the density of the fluid is a constant. Therefore, Equation (4) can be simplified as follows:

(5) $$\frac{𝜕\left(\rho u_{x}\right)}{𝜕x}+\frac{𝜕\left(\rho u_{y}\right)}{𝜕y}+\frac{𝜕\left(\rho u_{z}\right)}{𝜕z}=0$$

2.  The Momentum Conservation Equation

The momentum conservation equation, also known as the equation of motion, is the specific application of Newton’s second law to fluid mechanics. It establishes the relationships among the density, velocity, and pressure of an ideal fluid and external forces, that is, the rate of increase in the fluid momentum in a differential element is equal to the sum of various forces acting on the differential element. Based on this, the momentum equations of the fluid in the x, y, and z directions can be derived as follows:

(6) $$\rho \left(\frac{𝜕u_{x}}{𝜕t}+u_{x}\frac{𝜕u_{x}}{𝜕x}+u_{y}\frac{𝜕u_{x}}{𝜕y}+u_{z}\frac{𝜕u_{x}}{𝜕z}\right)=-\frac{𝜕p}{𝜕x}+\mu \left(\frac{{𝜕}^{2}u_{x}}{𝜕x^{2}}+\frac{{𝜕}^{2}u_{x}}{𝜕y^{2}}+\frac{{𝜕}^{2}u_{x}}{𝜕z^{2}}\right)+\rho f_{x}$$

(7) $$\rho \left(\frac{𝜕u_{y}}{𝜕t}+u_{x}\frac{𝜕u_{y}}{𝜕x}+u_{y}\frac{𝜕u_{y}}{𝜕y}+u_{z}\frac{𝜕u_{y}}{𝜕z}\right)=-\frac{𝜕p}{𝜕y}+\mu \left(\frac{{𝜕}^{2}u_{y}}{𝜕x^{2}}+\frac{{𝜕}^{2}u_{y}}{𝜕y^{2}}+\frac{{𝜕}^{2}u_{y}}{𝜕z^{2}}\right)+\rho f_{y}$$

(8) $$\rho \left(\frac{𝜕u_{z}}{𝜕t}+u_{x}\frac{𝜕u_{z}}{𝜕x}+u_{y}\frac{𝜕u_{z}}{𝜕y}+u_{z}\frac{𝜕u_{z}}{𝜕z}\right)=-\frac{𝜕p}{𝜕z}+\mu \left(\frac{{𝜕}^{2}u_{z}}{𝜕x^{2}}+\frac{{𝜕}^{2}u_{z}}{𝜕y^{2}}+\frac{{𝜕}^{2}u_{z}}{𝜕z^{2}}\right)+\rho f_{z}$$

In the formula, p represents the pressure on the differential element; μ represents the viscosity coefficient of the fluid; and $f_{x}$, $f_{y}$, $f_{z}$ are the body forces per unit mass in the three spatial directions.

3.  The Energy Conservation Equation

The energy conservation equation can be obtained according to the first law of thermodynamics. Its physical meaning is that the rate of increase in the thermodynamic energy within a differential element is equal to the sum of the net heat flow entering the differential element and the work done on the differential element by body forces and surface forces. Its governing equation is as follows:

(9) $$\frac{𝜕\left(\rho T\right)}{𝜕t}+div\left(\rho ut\right)=div\left(\frac{k}{C_{p}}grad\right)+S_{T}$$

Expand it and we obtain:

(10) $$\frac{𝜕\left(\rho T\right)}{𝜕t}+\frac{𝜕\left(\rho u_{x}T\right)}{𝜕x}+\frac{𝜕\left(\rho u_{y}T\right)}{𝜕y}+\frac{𝜕\left(\rho u_{z}T\right)}{𝜕z}=\frac{𝜕}{𝜕x}\left(\frac{k}{C_{p}}\frac{𝜕T}{𝜕x}\right)+\frac{𝜕}{𝜕y}\left(\frac{k}{C_{p}}\frac{𝜕T}{𝜕y}\right)+\frac{𝜕}{𝜕z}\left(\frac{k}{C_{p}}\frac{𝜕T}{𝜕z}\right)+S_{T}$$

In the formula, $C_{p}$ represents the specific heat capacity; k represents the heat transfer coefficient; T represents temperature; and $S_{T}$ represents the viscous dissipation term.

### 2.2. Establishment of the Nozzle Simulation Model

#### 2.2.1. Nozzle Geometric Model

**Figure 1:**
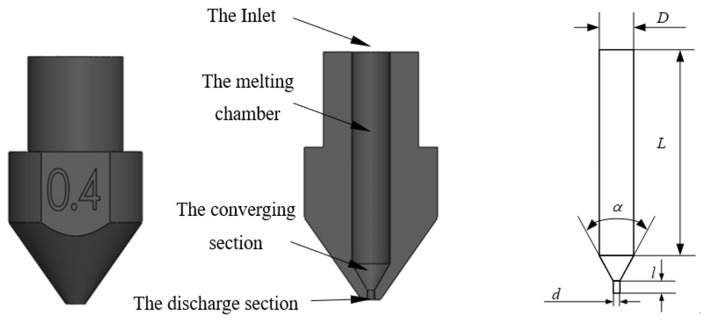
Three-dimensional printing nozzle model.

In order to understand the influence of the nozzle’s structural parameters on the melt extrusion speed, it was necessary to conduct simulations on the melt inside the nozzle. Firstly, a simulation model of the nozzle needed to be established. For the convenience of calculation, the threads were ignored and it was modeled using the SolidWorks 2021 3D software, as shown in Figure 1. The internal flow channel of the nozzle mainly consists of three parts: the melting chamber, the converging section, and the discharge section. Generally, it is described by the following structural parameters: the inlet diameter D, the length of the melting chamber L, the convergence angle α, the length l of the discharge section, and the inner diameter d of the nozzle.

#### 2.2.2. Mesh Generation, Material Model, and Boundary Conditions

1. Mesh Generation

**Figure 2:**
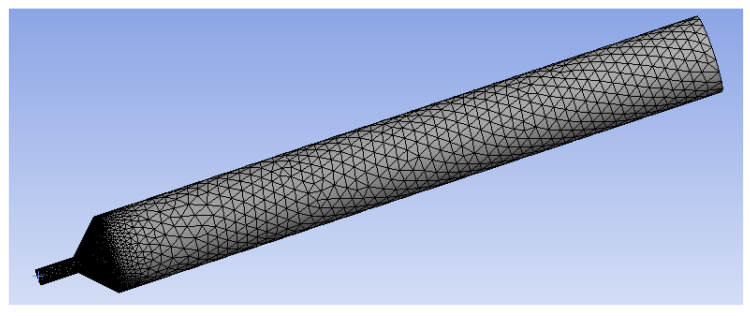
Mesh generation for the internal flow channel model of the nozzle.

It can be known from the above analysis that the flow field velocity of the melt is mainly related to the structural parameters of the internal flow channel of the nozzle. Therefore, only the three-dimensional model of the internal flow channel of the nozzle was meshed. In this paper, the meshing tool in the ANSYS Workbench (version 2021) platform is used. The mesh type was set as regular tetrahedrons. In order to make the convergence speed faster and improve the simulation calculation accuracy at the melt outlet, the mesh at the outlet was refined, as shown in Figure 2.

2.  Material Model

The polyether ether ketone (PEEK) material has non-Newtonian and shear-thinning properties, and its viscosity changes significantly during the melt extrusion process. Therefore, the change in viscosity needs to be taken into account during numerical simulations. Commonly used viscosity models include the power law model, the Cross–WLF model, and the Carreau–Yasuda model [31]. According to the principle of fused deposition modeling, there is a wide temperature variation range and relatively high pressure during the melt extrusion process of filaments. Hence, the seven-parameter Cross–WLF viscosity model is adopted in this paper. Its expressions are shown in Equations (11)–(14) as follows:

(11) $$\eta =\frac{\eta_{0}}{1+(\frac{\eta_{0}\dot{\gamma }}{\tau^{*}}{)}^{1-n}}$$

(12) $$\eta_{0}=D_{1}\exp\left[\frac{A_{1}\left(T-T^{*}\right)}{A_{2}+\left(T-T^{*}\right)}\right]$$

(13) $$A_{2}=A_{3}+D_{3}P$$

(14) $$T^{*}=D_{2}+D_{3}P$$

In the formula, η represents the viscosity of the PEEK material (Pa·s); $\eta_{0}$ represents the zero-shear viscosity; $\dot{\gamma }$ represents the shear rate ($s^{-1}$); $\tau^{*}$ represents the shear stress level at which the rheological characteristics of the melt transition from the Newtonian region to the power law region; n represents the flow index; T represents the current temperature of the melt (K); $T^{*}$ represents the reference temperature (K); P represents the current pressure of the melt (Pa); and $D_{1}$, $A_{1}$, $A_{3}$, $D_{3}$, and $D_{2}$ are model constants.

The parameters of the Cross–WLF viscosity model for the selected PEEK printing consumables are shown in Table 1.

3.  Boundary Conditions

According to the actual working conditions, the fluid part was defined as the PEEK material, and the specific parameters of the material are shown in Table 2. The filament inlet was defined as the fluid inlet boundary condition, with the inlet velocity set as the wire feeding speed of 2 mm/s and the inlet temperature as 143 °C [32]. The inner flow channel wall surface of the nozzle was defined as a constant wall surface, and the temperature was set as the printing temperature of 400 °C. The fluid outlet was defined as a pressure outlet, with the outlet pressure approximately equal to the standard atmospheric pressure and the outlet temperature set to 150 °C (which is the temperature inside the constant-temperature forming chamber).

### 2.3. Analysis of Simulation Results

#### 2.3.1. Analysis of the Distribution Law of the Melt Flow Field Velocity Within the Nozzle

**Figure 3:**
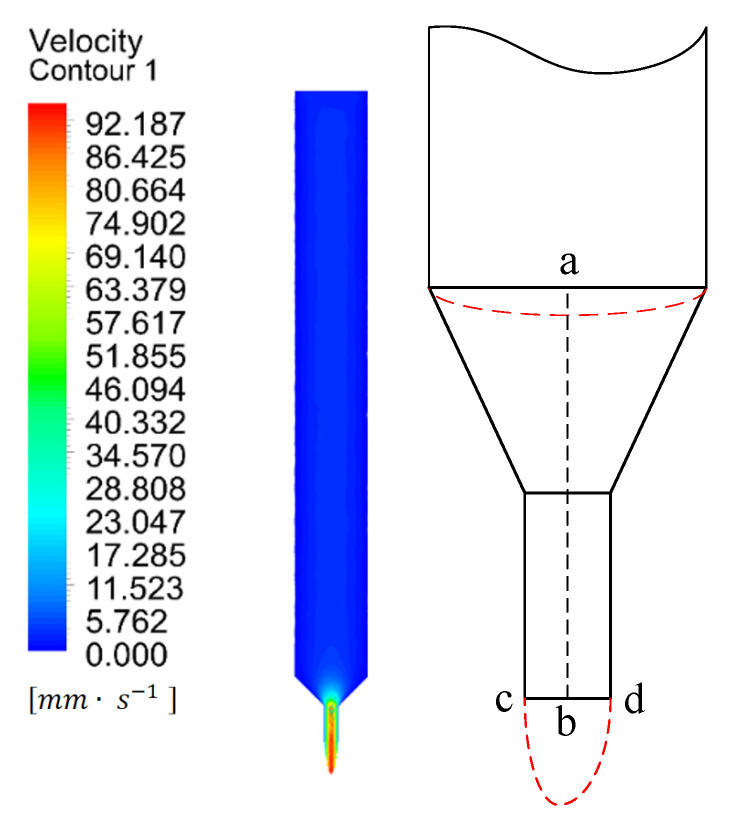
Melt velocity field inside the nozzle.

**Figure 4:**
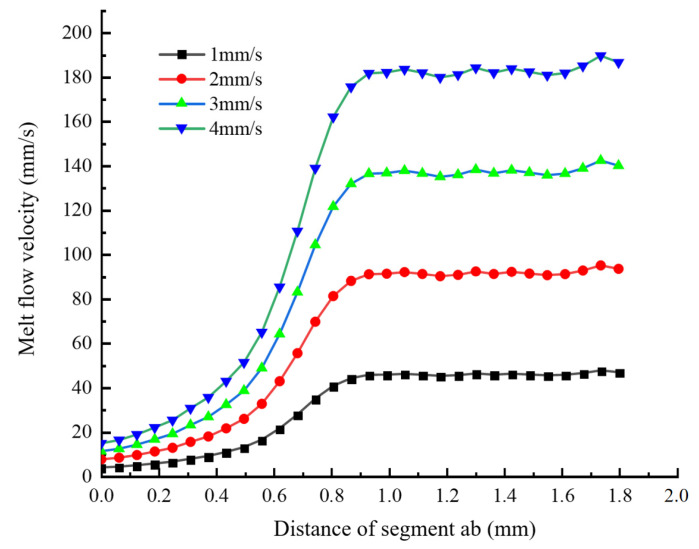
The trend of velocity change of the melt along segment ab under different wire feeding speeds.

**Figure 5:**
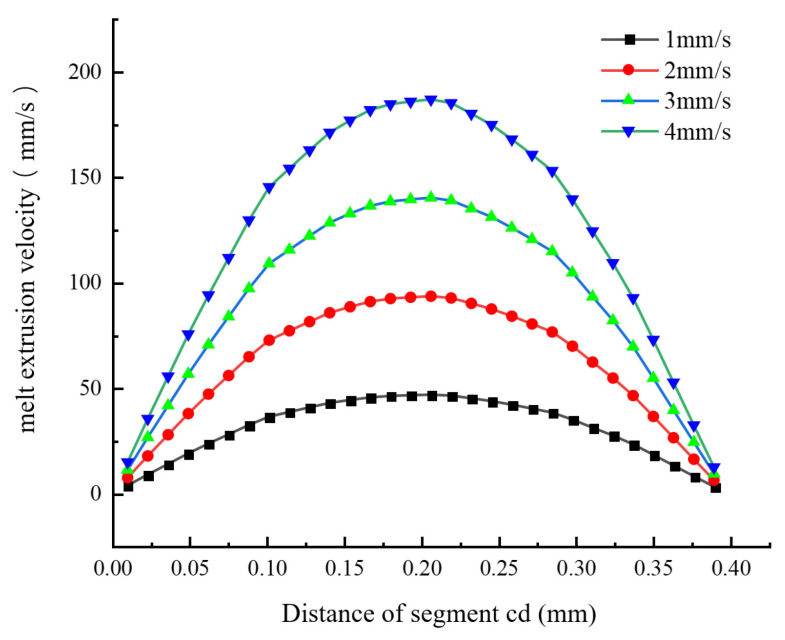
The trend of velocity change of the melt along segment cd under different wire feeding speeds.

Figure 3 shows the simulated melt velocity field under the conditions of the printing temperature (400 °C) and the wire feeding speed (2 mm/s). It can be seen that the melt flows in the melting chamber at an average speed of 5 mm/s, and the flow velocity of the melt presents a quasi-uniform distribution within the chamber. After the fluid enters the converging section, the flow velocity gradually increases and reaches its maximum when flowing out at the end of the converging section. When the fluid reaches the outlet, it gushes out in a convex shape, and the flow velocity near the pipe wall is less than the jet velocity near the pipe center. In order to display the respective laws of the melt velocity at the converging section and the outlet more clearly, a simulation model of the nozzle was constructed, as shown in Figure 4 and Figure 5. The segment ab represents the axial direction of the nozzle outlet, and the segment cd represents the radial direction of the nozzle outlet. Then, the velocity fields of these two different segments were further simulated.

1. Simulation Analysis of the Variation of Melt Velocity Along Segment ab

Figure 4 shows the changing trend of the melt flow velocity along segment ab under different wire feeding speeds (1 mm/s, 2 mm/s, 3 mm/s, and 4 mm/s). It can be seen that the changing trends of the melt flow velocity in the axial direction of the nozzle outlet under different wire feeding speeds are similar. They all rise first, then reach the maximum velocity range, change within this range, and, finally, tend to be stable. In other words, the melt velocity increases rapidly within the converging section of the nozzle and reaches the maximum level after moving a certain distance along the outlet. As the wire feeding speed increases (by 1 mm/s), the velocity at the inlet of the converging section increases steadily at a rate of 3.6 mm/s and reaches the maximum value (14.52 mm/s) when the wire feeding speed is 4 mm/s. Under different wire feeding speeds, the maximum extrusion velocities of the melt are 46.66 mm/s, 93.31 mm/s, 139.97 mm/s, and 186.62 mm/s, respectively. When the wire feeding speed increases by 1 mm/s, the maximum extrusion velocity of the melt increases steadily at a rate of 46.65 mm/s. This indicates that the extrusion velocity of the melt increases linearly with the linear increase of the wire feeding speed, also conforming to the pipe flow formula $A_{1}v_{1}=A_{2}v_{2}=Q$. It can be known that, when the flow rate is fixed and the inlet velocity and cross-sectional area are certain, the smaller the outlet cross-sectional area, the faster the outflow velocity.

2.  Simulation Analysis of the Variation of Melt Velocity Along Segment cd

Figure 5 shows the changing trend of the melt flow velocity along segment cd under different wire feeding speeds (1 mm/s, 2 mm/s, 3 mm/s, and 4 mm/s). It can be seen from Figure 5 that, under different wire feeding speeds, the changing trends of the melt extrusion velocity in the radial direction of the nozzle outlet are similar, showing a Gaussian distribution. Due to the existence of the viscous effect of the liquid and the wall friction, a velocity boundary layer appears near the wall. When the wire feeding speed is 1 mm/s, the extrusion velocity near the center of the outlet cross-section is relatively large, and, as the radial distance (that is, the distance from the outlet center line) increases, the extrusion velocity gradually decreases. However, as the wire feeding speed increases, the gradient of the radial velocity change of the melt gradually becomes larger, mainly because the increase in velocity leads to an increase in pressure drop. Generally speaking, the jet velocity in the center of the nozzle outlet is the largest, and the jet velocity near the pipe wall is the smallest. In addition, as the wire feeding speed increases, the gradient of the change in the melt extrusion velocity at the center of the nozzle outlet gradually increases.

For nozzles, the converging section and the discharging section determine the melt extrusion velocity at the nozzle outlet. In other words, the convergence angle α, the length l of the discharging section, and the inner diameter d of the nozzle are the main structural parameters that influence the melt extrusion velocity. The parameter C (the length-to-diameter ratio of the nozzle’s discharging section) is defined as the ratio of the length l of the discharging section to the inner diameter d of the nozzle, that is, C=l/d. The influences of α and C on the velocity distribution in the flow field are studied in detail below.

#### 2.3.2. The Influence of the Nozzle Convergence Angle on the Velocity Distribution of the Flow Field

**Figure 6:**
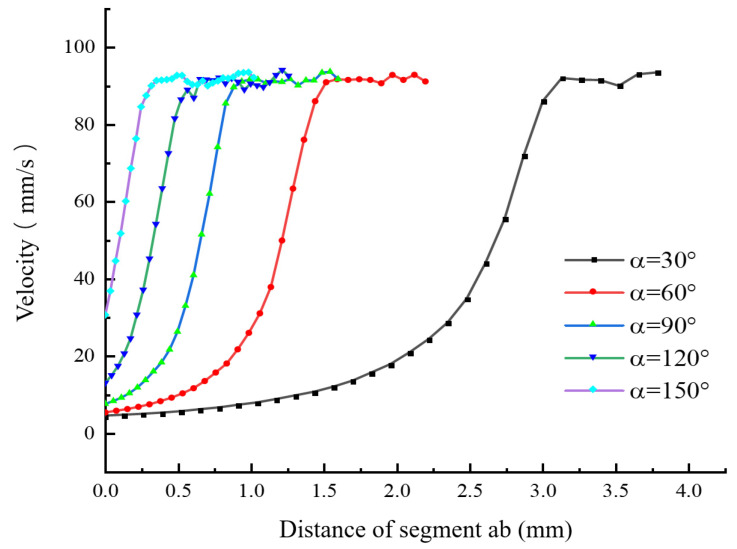
Velocity distribution of the melt along segment ab under different convergence angles.

While keeping the other parameters of the nozzle unchanged, numerical simulations were conducted on the nozzle fluidity under different convergence angles (α = 30°, 60°, 90°, 120°, and 150°), and the influence of the convergence angle on the melt flow velocity was obtained. Figure 6 present the simulation results of the flow velocity under different convergence angles.

**Figure 7:**
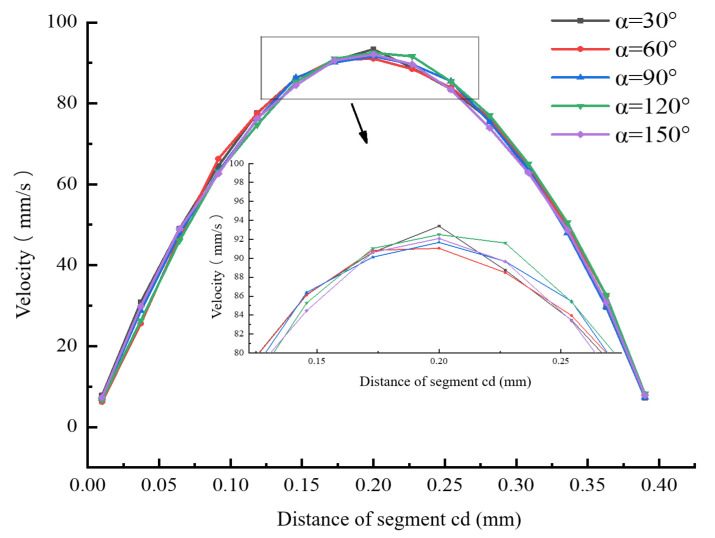
Velocity distribution of the melt along segment cd under different convergence angles.

**Figure 8:**
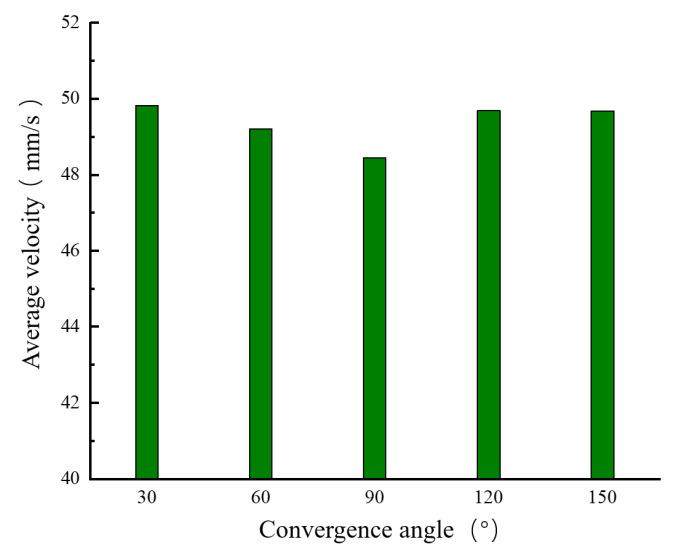
Histogram of the average velocity at the nozzle outlet cross-section under different convergence angles.

Figure 6 and Figure 7 show the velocity distributions along segment ab and segment cd under different convergence angles. It can be seen from these figures that, as the convergence angle α increases, the melt flow velocity near the convergence angle of segment ab increases at a faster rate. This is because the larger the convergence angle, the smaller the contact area between the melt and the wall surface, and, thus, the resistance that needs to be overcome between the melt and the wall surface becomes smaller. The curves of the melt flow velocity change in segment cd are similar. When α = 120°, the velocity in the center of the nozzle outlet is relatively high, and the velocity change toward both sides is more symmetrical. This can ensure the directional accuracy when the melt is extruded and further guarantee the dimensional precision of the printed model. Although when α = 150° the melt flow velocity near the convergence angle of segment ab increases at its fastest, this leads to a smaller area in the converging section and easily causes the melt to clog at the nozzle outlet. Figure 8 shows the distribution of the average extrusion velocity under different convergence angles. It can be seen from the figure that, when α = 90°, the average extrusion velocity of the melt is at its smallest, and the average velocities under the other four different convergence angles are basically the same. To sum up, when α = 120°, the radial velocity change is smaller and more symmetrical, and the distribution is relatively concentrated near the nozzle outlet. Therefore, it can be inferred that, when α = 120°, the melt flow effect is optimal.

#### 2.3.3. The Influence of the Length-to-Diameter Ratio of the Nozzle’s Discharging Section on the Velocity Distribution in the Flow Field

**Figure 9:**
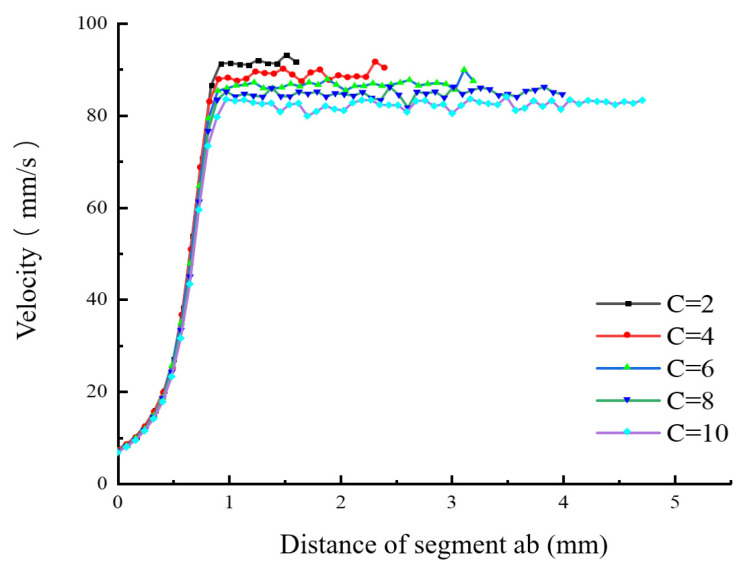
Velocity distribution of the melt along segment ab under different length-to-diameter ratios.

**Figure 10:**
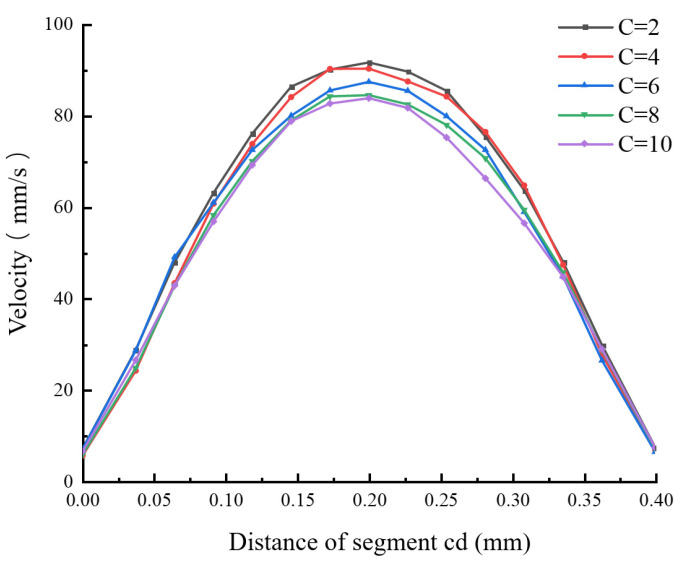
Velocity distribution of the melt along segment cd under different length-to-diameter ratios.

**Figure 11:**
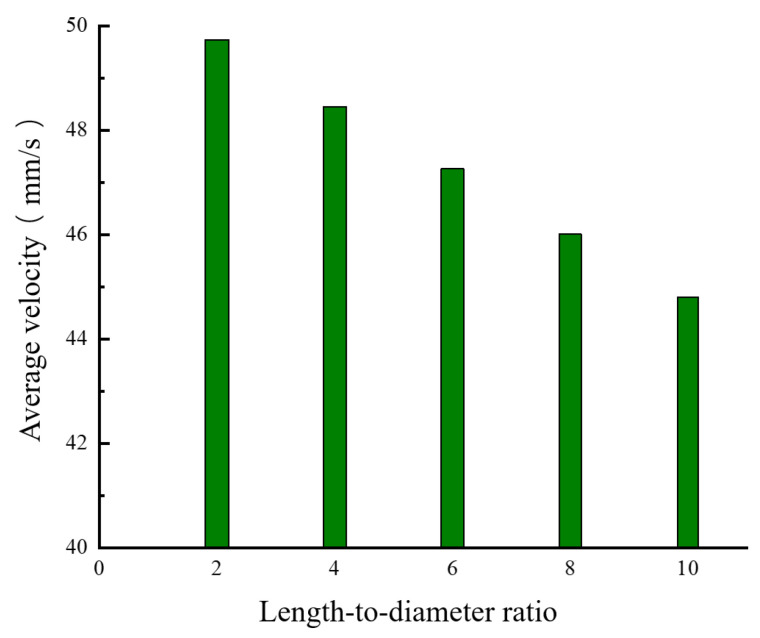
Histogram of the average velocity at the nozzle outlet cross-section under different length-to-diameter ratios.

While keeping the other parameters of the nozzle unchanged, numerical simulations were conducted on the nozzle fluidity under different length-to-diameter ratios (C = 2, 4, 6, 8, and 10), and the influence of the length-to-diameter ratio on the melt flow velocity was obtained. Figure 9, Figure 10 and Figure 11 show the simulation results of the flow velocity under different length-to-diameter ratios.

Figure 9 and Figure 10 show the velocity distributions of the melt along segment ab and segment cd under different length-to-diameter ratios. It can be clearly seen that, as the length-to-diameter ratio increases, the flow velocity of the melt gradually decreases, indicating that, when the length l of the discharging section is relatively long, the melt velocity increases more slowly. This is because, when the length l of the discharging section is relatively long, the pressure difference within the discharging section is reduced and the resistance of the fluid that needs to be overcome increases, thus reducing the melt flow velocity. In the axial direction, as C increases, the melt flow velocity near the nozzle outlet first increases and then tends to stabilize within a very small velocity range. When nozzles with different length-to-diameter ratios are used, the variation laws of the velocity of the melt along segment cd are similar, and the melt flow velocity is at its fastest near the center of the nozzle. As the radial distance increases, the flow velocity gradually decreases. Figure 11 is a histogram of the distribution of the average extrusion velocity under different length-to-diameter ratios. It can be seen from the figure that, as the length-to-diameter ratio C increases, the average velocity at which the melt is extruded from the nozzle gradually decreases and reaches its maximum when C = 2. Moreover, the change in the value of C has little impact on the flow velocity in the radial direction (segment cd), indicating that the nozzle achieves the optimal extrusion velocity when the length-to-diameter ratio C is 2.

### 2.4. Simulation Verification

**Figure 12:**
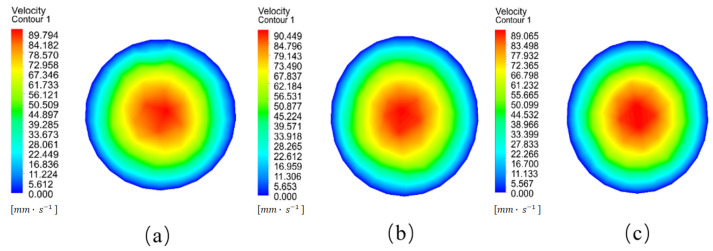
Velocity contour diagrams of the nozzle outlet cross-section before and after optimization. (**a**) Unoptimized; (**b**) optimized convergence angle, α = 120°; (**c**) optimized length-to-diameter ratio, C = 2.

The nozzles, before and after optimization, were imported into the ANSYS Fluent software (version 2021) for fluid simulations. The pre-processing conditions for the fluid analysis were kept unchanged. The velocity contour diagrams of the melt at the nozzle outlet cross-section before and after optimization are shown in Figure 12. The CFD Post tool in the Workbench platform was used to calculate the average value of the velocity at the nozzle outlet cross-section. The results are shown in Table 3.

It can be seen from Figure 12 that the velocity distribution at the center of the optimized nozzle is more uniform and has a wider range compared to that before optimization. As shown in Table 3, the average velocity at the nozzle outlet cross-section is significantly improved after optimization. The nozzles with the optimized convergence angle and length-to-diameter ratio have an increase in the velocity at the outlet cross-section of 2.5% and 2.7%, respectively. This enables the melt to be extruded from the nozzle more smoothly, improves the printing efficiency, reduces the probability of nozzle clogging during the printing process, and effectively ensures the surface quality and dimensional precision of the printed product. Therefore, it can be concluded that the melt extrusion velocity within the nozzle is optimal when the convergence angle is 120° and the length-to-diameter ratio is 2. This type of nozzle was selected to prepare for the subsequent experiments.

## 3. The Influence of Forming Process Parameters on Forming Accuracy and Optimization

The dimensional accuracy of the formed parts in fused deposition modeling (FDM) is an important indicator for measuring the quality of the formed parts. During the FDM process, different process parameters have varying degrees of influence on the forming accuracy. In this paper, the self-designed FDM equipment is used as an experimental tool. The orthogonal experimental method was adopted to analyze the influence of important process parameters on the dimensional accuracy of the formed parts. The optimal combination of process parameters was obtained through the comprehensive weighted scoring method and verified by printing tests.

### 3.1. Analysis of the Influencing Factors of Forming Accuracy

The process parameters of the fused deposition modeling (FDM) generally include printing speed, layer thickness, nozzle temperature, heated bed temperature, filling pattern, filling rate, and so on. Different process parameters have different influences on the dimensional accuracy of the formed parts. Based on the nozzles with the optimized convergence angle and length-to-diameter ratio in the previous section, combined with practical engineering experience and the analysis of the principle of fused deposition modeling, the factors that had a relatively large impact on the forming accuracy were selected as follows.

Printing speed: it refers to the scanning speed of the printing nozzle when depositing materials on the XOY plane. When the printing speed is too high, on the one hand, the vibration of the equipment increases and the moving inertia of the nozzle assembly increases, resulting in a reduction in the accuracy during the reciprocating movement process. On the other hand, it makes the feeding speed no match for the printing speed. After the melt is extruded from the nozzle, it is stretched into filaments as the nozzle moves, and even the “filament breakage” phenomenon may occur, causing insufficient filling of the model. When the printing speed is too slow, the melt is likely to adhere to the nozzle to form nodules, and, over time, the nozzle is likely to become clogged.

Layer thickness: it represents the thickness of each printed layer. It is equivalent to the vertical resolution of FDM parts. The thinner the layers, the smoother the texture of the printed formed parts, but more printing time will be required. The setting of the layer thickness is related to the inner diameter of the nozzle in use, and it is usually set to 20–80% of the inner diameter of the nozzle.

Nozzle temperature: it refers to the heating temperature of the consumables in the extrusion head. The setting of the temperature parameter affects the viscosity of the consumables. If the temperature is set too low, the consumables will not be fully melted, the melt viscosity will be relatively high, the extrusion will be discontinuous, and it is easy to cause the nozzle to become blocked by materials. If the temperature is set too high, the extruded molten consumables will be close to the liquid state, resulting in a decrease in the melt viscosity coefficient, making it difficult to accumulate and form and increasing the dimensional error of the formed parts.

Filling rate: it refers to the filling rate inside the formed model. The filling rate directly affects the performance of the formed parts. When the filling rate is relatively high, better mechanical properties can be achieved, but more time and consumables will be consumed, and even the phenomenon of “lumping” may occur due to the overly dense arrangement of the filaments in the formed layers. When the filling rate is relatively low, the forming efficiency becomes higher, but the mechanical performance is reduced, and depressions may appear on the upper end surface of models with smaller forming angles.

### 3.2. Experimental Design

#### 3.2.1. Setup of Experimental Equipment

**Figure 13:**
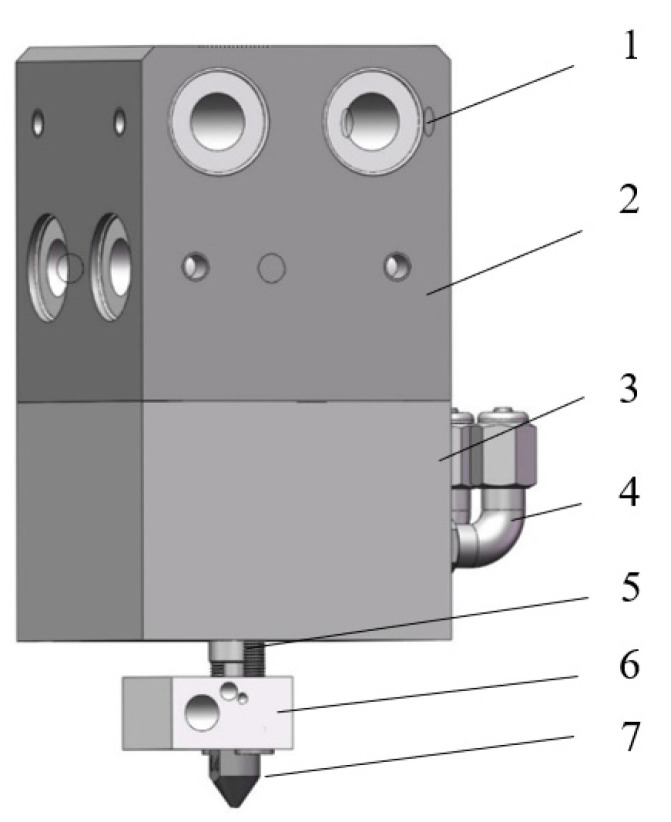
Nozzle assembly: 1. linear bearing; 2. four-way connecting block; 3. water cooling head; 4. quick-coupling elbow; 5. throat pipe; 6. heating block; 7. nozzle.

**Figure 14:**
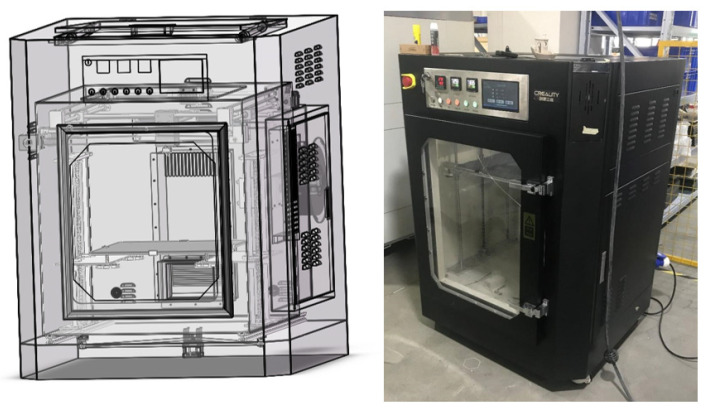
Virtual diagram of the whole machine assembly and the actual object.

Based on the optimized design of the nozzle in Section 2, on the basis of the existing Sermoon-M1 3D printer (Shenzhen Creality 3D Technology Co., Ltd., Shenzhen, China) in the laboratory, a nozzle with a convergence angle of 120° and a length-to-diameter ratio of 2 was adopted, and the printing nozzle assembly was independently designed. The nozzle assembly is shown in Figure 13. Layout design and assembly were carried out for all the components. With the help of the SolidWorks 3D software, the virtual assembly of the equipment was completed to ensure that there was no interference in the assembly and feasibility of the actual assembly. Then, the required parts were purchased, the non-standard parts were processed and manufactured, and the physical construction of the 3D printer was completed. The virtual assembly diagram and the physical diagram are shown in Figure 14.

#### 3.2.2. Experimental Model Design

**Figure 15:**
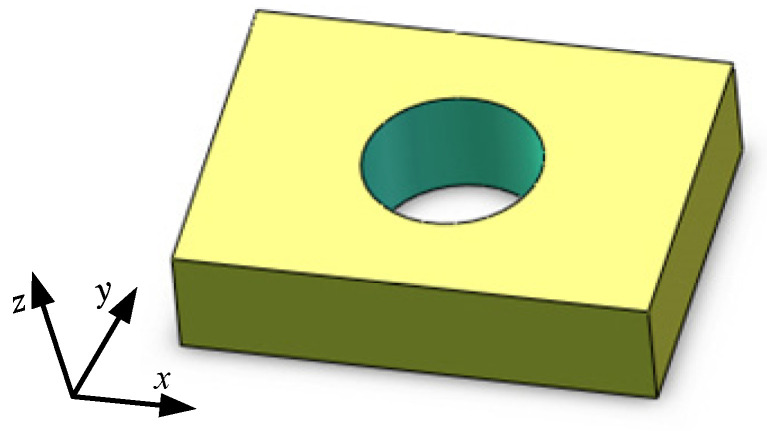
Test sample model.

In order to better reflect the geometric characteristics and facilitate measurements, a cuboid with a cylindrical hole inside was designed and modeled in the SolidWorks 3D software, as shown in Figure 15. The dimension in the *x*-axis direction was 40 mm, that in the *y*-axis direction was 35 mm, and that in the *z*-axis direction was 10 mm. The diameter of the circular hole was 15 mm.

#### 3.2.3. Experimental Equipment

**Figure 16:**
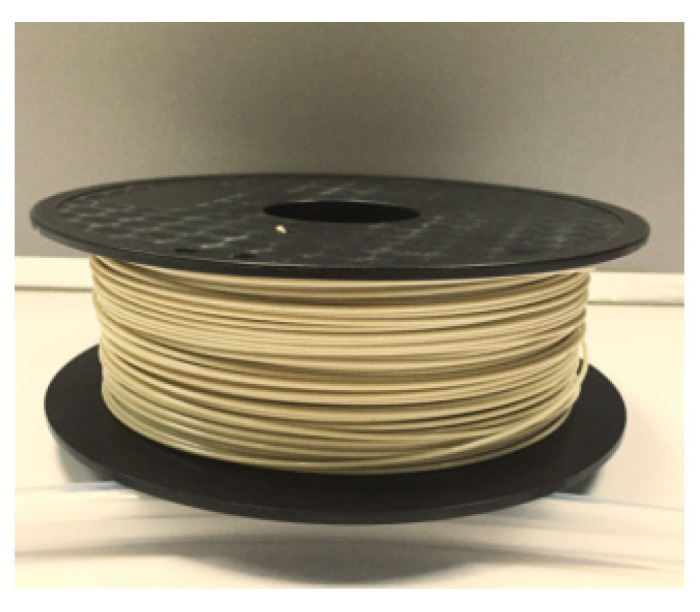
PEEK consumables.

**Figure 17:**
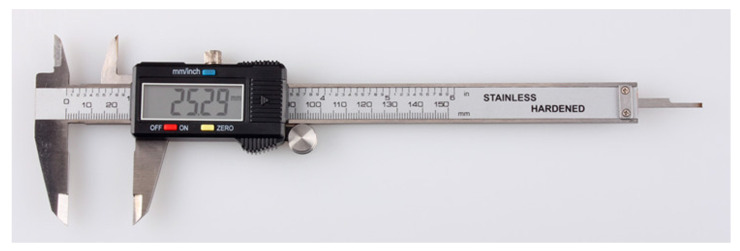
Digital display vernier caliper.

The experimental equipment adopted in this experiment was the Sermoon-M1 3D printer with an optimized nozzle structure. The printing consumable was PEEK material as shown in Figure 16. The measuring tool was a digital display vernier caliper with a measuring accuracy of 0.01 mm which was used to measure the dimensions in the x, y, and z directions as well as the diameter of the circular hole of the test samples under each test condition as shown in Figure 17. 

#### 3.2.4. Orthogonal Experimental Design

Orthogonal experiments can determine the influence laws of various factors on the experimental indexes with a relatively small number of experimental runs. It can tell which factors have a major influence, which ones have a minor influence, and which factors have mutual influences. Eventually, a combination of levels for each factor can be selected to determine the optimal process conditions [33].

In this experiment, the dimensional errors in the x, y, and z directions of the test pieces and the diameter error of the circular hole were taken as the optimization indexes. According to the influencing factors determined in Section 3.1, there were a total of four influencing factors in this orthogonal experiment, namely the printing speed $v\;\left(A\right)$, the layer thickness $h\;\left(B\right)$, the nozzle temperature $T\;\left(C\right)$, and the filling rate $\beta \;\left(D\right)$. Three levels were designed for each factor, respectively, and the results are shown in Table 4.

According to the designed factors and levels table, the $L9\left(3^{4}\right)$ orthogonal array was adopted, and a total of nine experiments were conducted. The specific experimental scheme is shown in Table 5.

### 3.3. Experimental Results and Analysis

#### 3.3.1. Experimental Results

**Figure 18:**
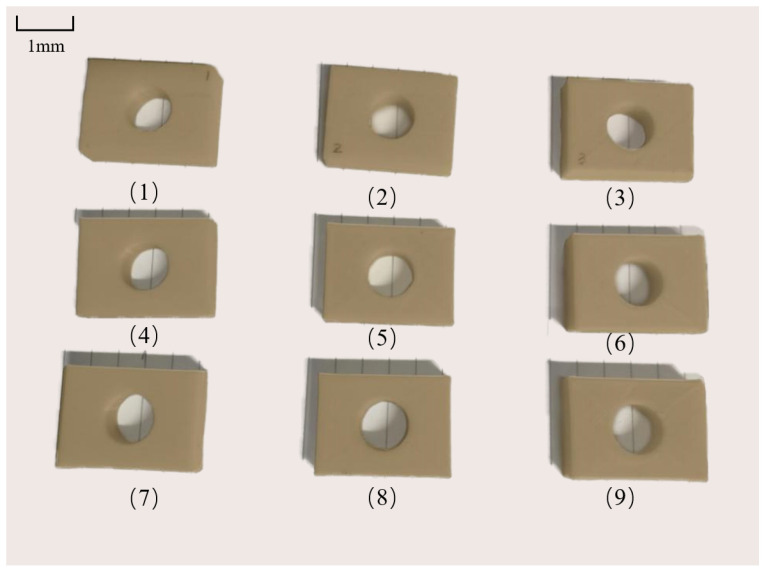
Test samples.

The printing parameters in the Ultimaker Cura 4.8 slicing software were set according to the process parameters in Table 5. The other process parameters were kept unchanged. The assembled experimental prototype was used to print the samples, and the test samples shown in Figure 18 were obtained. Then, the dimensions in the x, y, and z directions as well as the diameter of the circular hole of these nine samples were measured. Measurements were taken three times each time and the average value was calculated. Also, the absolute values of the dimensional errors in the three directions and the error of the circular hole diameter were calculated. The results of the orthogonal experiment are shown in Table 6.

#### 3.3.2. Range Analysis

Range analysis is an intuitive analytical method, also known as the R method. It judges the superiority and inferiority of factors by calculating the R value (the range value of factors) and can also determine the optimal level under a certain factor, so as to obtain the final combination. When conducting range analysis, first calculate the $K_{i}$ value and $k_{i}$ value of the index statistical parameters under each level of the experimental factors, and then calculate the range $R_{j}$ value [34]:

(15) $$K_{i}=\overset{n}{\underset{k=1}{\sum }}Y_{k}$$

(16) $$k_{i}=\frac{1}{n}K_{i}$$

(17) $$R_{j}=\max\left\{\begin{array}{ccc} k_{1}, & k_{2}, & \ldots \end{array}\right\}-\min\left\{\begin{array}{ccc} k_{1}, & k_{2}, & \ldots \end{array}\right\}$$

In the formula, $K_{i}$ represents the total value of the results of the experimental factor at the i level; $Y_{k}$ represents the value of the kth index; $k_{i}$ is the average value of $k_{i}$; and n is the number of experimental levels.

**Figure 19:**
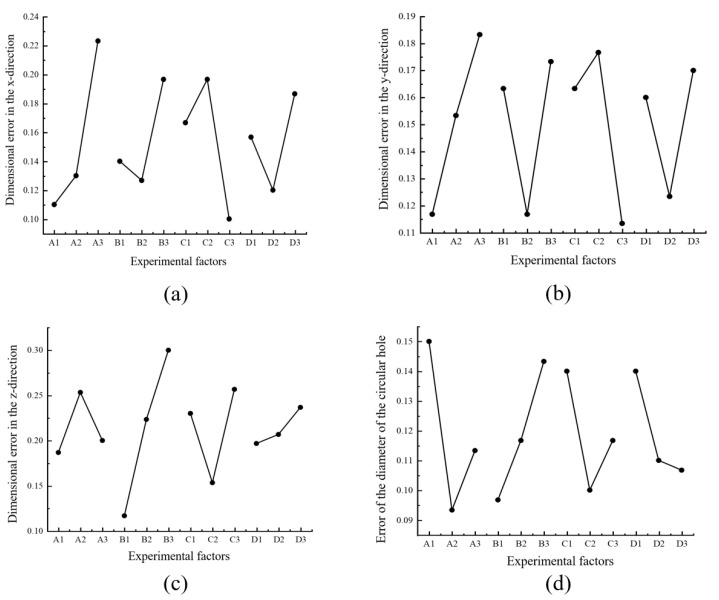
Curves of the relationship between each factor level and the evaluation indexes: (**a**) relationship between each factor and the dimension index in the x-direction; (**b**) relationship between each factor and the dimension index in the y-direction; (**c**) relationship between each factor and the dimension index in the *z*-direction; (**d**) relationship between each factor and the circular hole diameter index.

Calculate the range values of each index according to the above formula; the results are shown in Table 7, Table 8, Table 9 and Table 10. To display the influence of each factor level on the evaluation indexes more intuitively, take the factor levels as the abscissa and each evaluation index as the ordinate, and the relationship diagrams between each factor level and the evaluation indexes, as shown in Figure 19, are obtained.

The following conclusions can be drawn by analyzing Figure 19.

1. Influence of Printing Speed v (Factor A) on Forming Accuracy

The range of printing speed in the dimensions of the x and y directions and the diameter of the circular hole were relatively large, indicating that the printing speed has a significant impact on the dimensions in the x and y directions and the diameter of the circular hole. Among them, as the printing speed increased, the dimensional errors in the x and y directions both increased, and the errors in these two directions were the largest when the printing speed was 45 mm/s. While the error of the circular hole diameter first decreased and then increased as the printing speed increased, when it was at A_2_ (printing speed of 30 mm/s), the error of the circular hole diameter was the smallest. The dimensional error in the z direction first increased and then decreased as the printing speed increased, and the error was the largest when the printing speed was 30 mm/s. It can be seen that reducing the printing speed can effectively improve the printing accuracy, but it consumes a lot of time in the printing manufacturing process. If the quality requirements for the printed parts are not high, the printing speed can be appropriately increased.

2.  Influence of Layer Thickness h (Factor B) on Forming Accuracy

The range of layer thickness in the dimension of the z direction was relatively large, indicating that the layer thickness has a significant impact on the dimension in the z direction. As the layer thickness increased, the dimensional error in this direction gradually became larger. As it can be seen from Figure 19c, it shows a nearly linear increasing relationship with the change in layer thickness. The variation laws of the dimensional errors in the x and y directions were consistent. As the layer thickness increased, they both first decreased and then increased. The variation law of the error of the circular hole diameter was similar to that of the dimensional error in the z direction. However, the range of change in the value of the error of the circular hole diameter was very small. A smaller layer thickness can make each layer stack more closely and bond better, which is beneficial for improving the mechanical properties of the printed workpiece and can also better ensure the dimensional accuracy in the z direction. However, it is also easy to cause the molten filament to be pressed flatter, which is not friendly to the dimensional accuracy in other directions. On the other hand, it also increases the required printing time.

3.  Influence of Nozzle Temperature T (Factor C) on Forming Accuracy

The range of nozzle temperature in the dimensions of the x and y directions was relatively large, indicating that the nozzle temperature has a significant impact on the dimensions in the x and y directions. The variation laws of the dimensional errors in the x and y directions and the error of the circular hole diameter were consistent. As the nozzle temperature rose, they all first increased and then decreased. The variation laws of the dimensional error in the z direction and the error of the circular hole diameter were also consistent, but they first decreased and then increased as the nozzle temperature rose. The nozzle temperature is set according to the selected consumables and is responsible for melting the filament materials. If the nozzle temperature is too high, the viscosity of the melt will be reduced, making it difficult to stack and form. If the temperature is too low, the printing consumables will not be fully melted in the nozzle, resulting in the extruded filament being too thin or in the extrusion not being smooth, so that the extruded filaments cannot be closely arranged. All these situations can lead to a reduction in the forming accuracy of the workpiece.

4.  Influence of Infill Rate β (Factor D) on Forming Accuracy

The ranges of the infill rate were all relatively small, indicating that it has a relatively small impact on each evaluation index. As the infill rate increased, the dimensional errors in the x and y directions both first decreased and then increased. For the change in the dimensional error in the z direction, it gradually increased as the infill rate increased, while the error of the circular hole diameter gradually decreased.

By analyzing the range values under each index, the order of the influence of the four factors on the dimensional error index from strong to weak is arranged as follows: for the dimension in the x direction: A > C > B > D; for the dimension in the y direction: A > C > B > D; for the dimension in the z direction: B > C > A > D; for the diameter of the circular hole: A > B > C > D.

#### 3.3.3. Process Parameter Optimization

It can be known from the range analysis above that the influence intensities of process parameters on each index were different, and the combinations of optimized process parameters under a single index were also inconsistent. Therefore, in this paper, based on the fact that, in actual printing, requirements are generally imposed on all directions of the printed workpiece, the comprehensive weighted scoring method [35] is adopted to convert multi-index optimization into single-index optimization and determine the optimal combination of process parameters.

Firstly, to ensure the reliability of the analysis results, dimensionless processing was carried out on each error of the orthogonal experiment. Moreover, for these four indexes, the smaller the values, the better. Therefore, the smaller-the-better characteristic [36] was adopted for data processing. Its calculation formula is as follows:

(18) $$X_{ij}^{*}=\frac{\underset{j}{\max}X_{ij}-X_{ij}}{\underset{j}{\max}X_{ij}-\underset{j}{\min}X_{ij}}\left(i=1,2,\ldots ,m;j=1,2,\ldots ,n\right)$$

In the formula, $X_{ij}^{*}$ is the sequence generated by the dimensionless parameters of the index variable; $X_{ij}$ is the experimental data of the index variable; m is the serial number, and, in this experiment, m took a value of 12; n is the number of indexes, and it took a value of 4.

Then, based on the data after dimensionless transformation and combined with the weights of each index in the forming accuracy requirements, the comprehensive weighted score was calculated. The calculation formula is as follows:

(19) $$F_{i}=\overset{n}{\underset{j=1}{\sum }}\omega_{j}X_{ij}^{*}$$

In the formula, $\omega_{j}$ represents the weight of the j-th dimensional index. In this experiment, it is considered that the accuracy requirements for the dimensions in the x, y, and z directions, as well as the diameter of the circular hole, are equally important, so $\omega_{j}=1/4$.

Finally, the above formula was used to calculate the dimensionless data and the comprehensive weighted scores, and the results are shown in Table 11.

**Figure 20:**
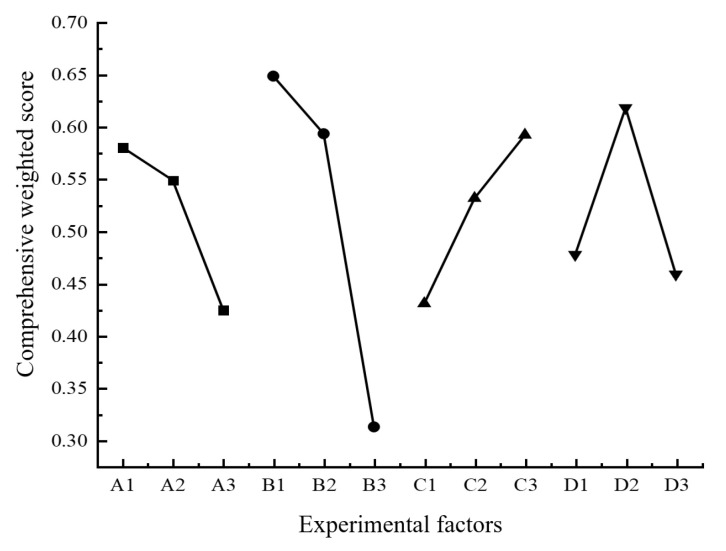
Relationship between the comprehensive weighted score and the levels of each factor.

Based on the comprehensive weighted scores in Table 11, the average values and the ranges of each level for the four factors were calculated. The results are shown in Table 12. Similarly, the relationship diagram between the comprehensive weighted scores and the levels of each factor was also drawn, as shown in Figure 20.

It can be seen from Table 13 that the ranges of the influencing factors A, B, C, and D were 0.1554, 0.3361, 0.1615, and 0.1595, respectively. Therefore, among these four factors, the factor that had the greatest impact on the forming accuracy was the layer thickness, followed by the infill rate, then by the nozzle temperature, and the printing speed had the smallest impact. It can be seen from Figure 20 that the variation laws of the printing speed and the layer thickness with respect to the comprehensive weighted score were consistent. As the printing speed and the layer thickness increased, the comprehensive weighted score first decreased slowly and then decreased sharply, and the change amplitude was the most significant under the factor of layer thickness. As the nozzle temperature rose, the comprehensive weighted score gradually increased. As the infill rate increased, the comprehensive weighted score first increased and then decreased, and, when it was at D_2_ (infill rate of 50%), the comprehensive weighted score reached its maximum value. To sum up, when the combination of process parameters is A_1_B_1_C_3_D_2_, the comprehensive weighted score is the theoretical maximum. That is, the process parameters are: the printing speed of 15 mm/s, the layer thickness of 0.1 mm, the nozzle temperature of 420 °C, and the infill rate of 50%.

### 3.4. Experimental Verification

The optimal process combination obtained through the data analysis in the previous text was A_1_B_1_C_3_D_2_, and this combination did not appear in the orthogonal experiment scheme. Therefore, the process parameters were set with a printing speed of 15 mm/s, a layer thickness of 0.1 mm, a nozzle temperature of 420 °C, and an infill rate of 50% to conduct a printing experiment. The measurement results are shown in Table 13, and a set of process parameters (A_2_B_2_C_1_D_1_) before optimization was selected as a reference for comparison.

It can be seen from Table 13 that the error of each index after optimization was smaller than that before optimization. However, compared with Table 6 of the orthogonal experiment results in the previous text, the errors after optimization were not all smaller than the error values under each index of the orthogonal experiment results. This is because the combination of process parameters in this group was optimized for multiple indexes. Therefore, the comprehensive weighted scores were calculated again for these two groups of data together with the previous orthogonal experiment data. It is concluded that the comprehensive weighted scores before and after optimization were 0.7693 and 0.9231, respectively, and the comprehensive weighted score after optimization was the highest, thus verifying the effectiveness of the optimization of this group of process parameters.

## 4. Conclusions

This paper aims to analyze the influence of the convergence angle and the length-diameter ratio of the nozzle of a 3D printer on the velocity of the melt inside the nozzle, determine the optimal nozzle structure, independently design the printing nozzle assembly, and apply it to the Sermoon-M1 3D printer to conduct experiments for determining the optimal process parameters. The specific contents are as follows:

1. Combined with thermal fluid simulations, the influence of the convergence angle and the length-diameter ratio in the nozzle structural parameters on the melt fluidity was explored. Through single-factor simulation analyses, it was concluded that the larger the convergence angle, the greater the change in the axial velocity in the convergence region, while the change in the radial velocity at the outlet was not significant. It was determined that, when the convergence angle was 120°, the melt flow effect was optimal. For nozzles with a relatively large length–diameter ratio, the increase rate of the melt along the axial direction of the outlet was slower, and the influence on the radial melt velocity was smaller. Similarly, it was also determined that, when the length–diameter ratio was 2, the melt extrusion effect was the best. Simulations were carried out on the nozzles before and after optimization, and the results showed that the nozzles with the optimized convergence angle and length–diameter ratio, respectively, increased the velocity at the outlet cross-section by 2.5% and 2.7%.
2. Orthogonal experiments were adopted to analyze the influence of the printing speed, layer thickness, nozzle temperature, and infill rate on the dimensional accuracy of the printed workpiece in the x, y, and z directions as well as of the diameter of the circular hole. The conclusion was drawn that the order of the influence intensity of each factor on the dimensional accuracy of the test samples in the x and y directions was the same (printing speed > nozzle temperature > layer thickness > infill rate), and the preferable test conditions were also the same (a printing speed of 15 mm/s, a layer thickness of 0.2 mm, a nozzle temperature of 420 °C, and an infill rate of 50%). The layer thickness had the most significant influence on the dimensional accuracy in the z direction, and the infill rate had a relatively small influence on the forming accuracy.
3. The comprehensive weighted scoring method was used to optimize the process parameters for multiple indexes, and a set of optimal process combinations (a printing speed of 15 mm/s, a layer thickness of 0.1 mm, a nozzle temperature of 420 °C, and an infill rate of 50%) was obtained. The effectiveness of the optimized process parameters was verified through experiments.

## Figures and Tables

**Table 1:** Parameters of the Cross–WLF viscosity model for PEEK printing consumables.

*n*	τ*/Pa	*D*_1_/(Pa·s)	*D*_2_/K	*D*_3_/(K·Pa)	*A*_1_	*A*_3_/K
0.4266	124,139	2.9 × 10^16^	403.15	0	36.041	51.6

**Table 2:** Related parameters of the polyether ether ketone (PEEK) material.

Property	Unit	Value
Density	g/cm^3^	1.31
Poisson’s ratio	—	0.4
Elastic modulus	Mpa	4400
Specific heat capacity	J/(kg·m)	2200
Thermal conductivity	W/(k·m)	0.25
Coefficient of thermal expansion	—	4.7 × 10^−5^

**Table 3:** Average velocity at the nozzle outlet cross-section before and after optimization.

Optimization Object	Before Optimization	After Optimization	Improvement Rate
Convergence angle	48.4507 mm/s	49.6856 mm/s	2.5%
Length-to-diameter ratio	48.4507 mm/s	49.7354 mm/s	2.7%

**Table 4:** Orthogonal experimental factors and levels table of FDM process parameters.

Levels	Experimental Factors			
	A/(mm/s)	B/mm	C/°C	D/%
1	15	0.1	400	20
2	30	0.2	410	50
3	45	0.3	420	80

**Table 5:** Orthogonal experiment scheme table.

Serial Number	Experimental Factors			
	A/(mm/s)	B/(mm)	C/(°C)	D/%
1	15	0.1	400	20
2	15	0.2	410	50
3	15	0.3	420	80
4	30	0.1	410	80
5	30	0.2	420	20
6	30	0.3	400	50
7	45	0.1	420	50
8	45	0.2	400	80
9	45	0.3	410	20

**Table 6:** Results of the orthogonal experiment.

Serial Number	Experimental Factors				Deviation			
	A	B	C	D	Δ*x*/mm	Δ*y*/mm	Δ*z*/mm	Δ*d*/mm
1	15	0.1	400	20	0.11	0.15	0.09	0.17
2	15	0.2	410	50	0.09	0.08	0.13	0.12
3	15	0.3	420	80	0.13	0.12	0.34	0.16
4	30	0.1	410	80	0.19	0.21	0.12	0.04
5	30	0.2	420	20	0.05	0.09	0.29	0.11
6	30	0.3	400	50	0.15	0.16	0.35	0.13
7	45	0.1	420	50	0.12	0.13	0.14	0.08
8	45	0.2	400	80	0.24	0.18	0.25	0.12
9	45	0.3	410	20	0.31	0.24	0.21	0.14

**Table 7:** Range results taking the dimension in the x-direction as an index.

	A	B	C	D
*K*_1_	0.3300	0.4200	0.5000	0.4700
*K*_2_	0.3900	0.3800	0.5900	0.3600
*K*_3_	0.6700	0.5900	0.3000	0.5600
*k*_1_	0.1100	0.1400	0.1667	0.1567
*k*_2_	0.1300	0.1267	0.1967	0.1200
*k*_3_	0.2233	0.1967	0.1000	0.1867
*R*	0.1133	0.0700	0.0967	0.0667

**Table 8:** Range results taking the dimension in the y-direction as an index.

	A	B	C	
*K*_1_	0.3500	0.4900	0.4900	0.4800
*K*_2_	0.4600	0.3500	0.5300	0.3700
*K*_3_	0.5500	0.5200	0.3400	0.5100
*k*_1_	0.1167	0.1633	0.1633	0.1600
*k*_2_	0.1533	0.1167	0.1767	0.1233
*k*_3_	0.1833	0.1733	0.1133	0.1700
*R*	0.0666	0.0566	0.0634	0.0467

**Table 9:** Range results taking the dimension in the z-direction as an index.

	A	B	C	
*K*_1_	0.5600	0.3500	0.6900	0.5900
*K*_2_	0.7600	0.6700	0.4600	0.6200
*K*_3_	0.6000	0.9000	0.7700	0.7100
*k*_1_	0.1867	0.1167	0.2300	0.1967
*k*_2_	0.2533	0.2233	0.1533	0.2067
*k*_3_	0.2000	0.3000	0.2567	0.2367
*R*	0.0666	0.1833	0.1034	0.0400

**Table 10:** Range results taking the diameter of the circular hole as an index.

	A	B	C	D
*K*_1_	0.4500	0.2900	0.4200	0.4200
*K*_2_	0.2800	0.3500	0.3000	0.3300
*K*_3_	0.3400	0.4300	0.3500	0.3200
*k*_1_	0.1500	0.0967	0.1400	0.1400
*k*_2_	0.0933	0.1167	0.1000	0.1100
*k*_3_	0.1133	0.1433	0.1167	0.1067
*R*	0.0567	0.0467	0.0400	0.0333

**Table 11:** Dimensionless data and comprehensive scores.

Serial Number	Dimensionless Data				Comprehensive Weighted Score
	*x* *	y *	z *	Diameter of the Circular Hole *	
1	0.7692	0.5625	1.0000	0.0000	0.5829
2	0.8462	1.0000	0.8462	0.3846	0.7692
3	0.6923	0.7500	0.0385	0.0769	0.3894
4	0.4615	0.1875	0.8846	1.0000	0.6334
5	1.0000	0.9375	0.2308	0.4615	0.6575
6	0.6154	0.5000	0.0000	0.3077	0.3558
7	0.7308	0.6875	0.8077	0.6923	0.7296
8	0.2692	0.3750	0.3846	0.3846	0.3534
9	0.0000	0.0000	0.5385	0.2308	0.1923

Note: * represents the sequences of the data of the 9 test specimens after dimensionless transformation in the four directions of x, y, z and the diameter of the circular hole.

**Table 12:** Range results of comprehensive weighted scores.

	A	B	C	D
*k*_1_	0.5805	0.6486	0.4307	0.4776
*k*_2_	0.5489	0.5934	0.5316	0.6182
*k*_3_	0.4251	0.3125	0.5922	0.4587
*R*	0.1554	0.3361	0.1615	0.1595

**Table 13:** Measurement results.

Combination of Process Parameters		Deviation			
		Δ*x*/mm	Δ*y*/mm	Δ*z*/mm	Δ*d*/mm
Before optimization	A_2_B_2_C_1_D_1_	0.10	0.13	0.06	0.09
After optimization	A_1_B_1_C_3_D_2_	0.07	0.08	0.05	0.07

## Data Availability

The original contributions presented in this study are included in the article. Further inquiries can be directed to the corresponding author.
